# Death of Prof. Charles Frick

**Published:** 1860-04

**Authors:** 


—It is with sincere regret that we announce the death of one of our friends, Prof. Charles Frick, of Baltimore, whose name is familiar to the profession throughout the country as an earnest student, and who, among his personal acquaintances, was universally esteemed as a true physician and a noble-hearted man. He died in the active duties of his profession, from diphtheria, claiming for himself, as a relief from suffering, the operation of tracheotomy, which he had himself performed upon a patient, at the Infirmary, but a few days before. The following just tribute to his memory we take from a Baltimore paper:

"Our readers, and the whole community, will share with us the poignant regret with which we are compelled to announce the unexpected and untimely death of Dr. Charles Frick, Professor of Materia Medica and Therapeutics in the University of Maryland, and one of our most promising and useful citizens. A malignant disease, contracted but a few days since, in the discharge of his professional duties at the Infirmary, was brought yesterday to a painful and melancholy close, before the tidings of his illness had reached more than a few of the large number by whom he was so justly admired and loved.

" Dr. Frick was a native of Baltimore, a son of the late Hon. William Frick, and at the time of his death had only reached his thirty-seventh year. Enthusiastically devoted to the profession of his choice, he had dedicated to its energetic pursuit all the faculties of an acute and admirable intellect, and had already attained a position in its ranks to which his election to the Chair he occupied was no more than a befitting testimonial. It is for those more familiar with the course of his varied scientific inquiries to render to his professional reputation the discriminating justice which, in so brief a notice as this, it would be wholly inappropriate to attempt. To the public at large he was known as a diligent and faithful student of books and of nature—an independent and original thinker, of large and careful observation and patient and laborious analysis. His contributions to medical literature were frequent and highly esteemed, and through them he was known most favorably to the Faculty, in Europe as well as his own country. In the practical department of his profession, he was eminently popular and successful, not less from the ability and conscientious care with which he fulfilled its laborious duties, than from his cordial and attractive manners and sympathizing nature. In the relations of private life no man ever gathered round him more unqualified affection and respect. He was full of gentleness and kindness, of modesty and unobtrusive charity. As was his work, so be his reward!"

